# Autophagy restricts HIV-1 infection

**DOI:** 10.18632/oncotarget.5123

**Published:** 2015-08-07

**Authors:** Coralie F. Daussy, Bruno Beaumelle, Lucile Espert

**Affiliations:** CPBS FRE 3689 CNRS – Université de Montpellier, Montpellier, France

**Keywords:** Autophagy Section, HIV, autophagy

Human Immunodeficiency Virus type 1 (HIV-1) is a retrovirus that targets cells from the immune system such as the CD4 T lymphocytes, the macrophages and the dendritic cells (DCs). Although these host cells possess various defense mechanisms to fight this infection, HIV-1 has evolved multiple strategies to counteract them. For the past ten years, researchers have been focused on the identification of HIV-1 restriction factors and their mode of action for the development of new therapeutic strategies [1].

Macroautophagy (hereafter referred to as autophagy) is a major cellular catabolic pathway involved in several fundamental physiological processes, such as homeostasis, innate and adaptive immunities, development and differentiation. It is characterized by the formation of vacuoles called autophagosomes that sequester cytoplasmic constituents. The sequestered material is then degraded and recycled after fusion of autophagosomes with lysosomes. The autophagic pathway is orchestrated by more than thirty specific proteins named ATGs acting at different levels of the process [2]. Autophagy is believed to be one of the most ancient mechanism of defense against intracellular pathogens. Consequently, pathogens evolved strategies to prevent or hijack this process to their own benefit. Autophagy has long been considered as a bulk degradation pathway but it becomes clear that it is a highly selective process. The selectivity of its substrates is mediated by adaptor proteins, such as p62/SQSTM1, NDP52, NBR1 or Optineurin, which interact with ubiquitinated proteins and organelles, targeting specific cargos to autophagosomes [2].

We have demonstrated that HIV-1 envelope glycoproteins (Env), trigger autophagy at the very first step of CD4 T cells infection by their interaction with specific receptors (i.e. CD4 and CCR5 or CXCR4). This autophagic process exerts a powerful anti-HIV effect by selectively degrading the viral transactivator Tat (Transactivator of Transcription), a viral protein absolutely required for the transcription of viral genes. The selectivity of this degradation is conferred by p62/SQSTM1, which interacts with Tat independently of its ubiquitination [3]. Beside its role in transcriptional elongation of HIV genes, Tat is also secreted by infected cells and can be captured by neighboring uninfected cells where it activates the transcription of different cellular genes or induces their death by apoptosis. Interestingly, incoming Tat can also be degraded by autophagy, expanding the potential role of this mechanism beyond the viral replication itself.

By selectively addressing Tat to lysosomal degradation, autophagy is a major anti-HIV pathway. The virus has thus evolved to avoid this powerful antiviral effect. Accordingly, HIV-1 is able to counteract autophagy at various levels by the action of several viral proteins, depending on the cellular context. For example, the HIV protein Vif, which interacts with the essential ATG protein MAP-LC3B (Microtubule Associated Protein-Light Chain 3B), has been shown to inhibit autophagy at the late steps of the viral replication cycle in CD4 T lymphocytes. Another HIV protein, Nef, is able to block the degradative steps of autophagy in human macrophages by interacting with the ATG protein Beclin 1. In the same cell type, Nef is also able to induce the cytoplasmic sequestration of the TFEB transcription factor, which is known to participate to the synthesis of ATGs. In DCs, autophagy is blocked by the HIV-1-Env-mediated Akt activation, likely avoiding the viral antigen presentation to CD4 T cells by the major histocompatibility complex [4]. The drugs currently used to treat HIV-infected patients target different steps of the viral cycle. Thus, stimulating the host response would be an added value to the already existing treatments. Within this context, it is tempting to speculate that new therapeutic agents that can induce autophagy could be beneficial for HIV-1-infected patients. Along these lines, we have shown that Torin-1, which induces autophagy by inhibiting mTOR, decreases the level of HIV-1 production in CD4 T lymphocytes. Rapamycin, which has the same mode of action and which is already clinically approved, has also been shown to possess anti-HIV properties both *in vitro* and *in vivo* [5]. Moreover, other strong inducers of autophagy such as vitamin D3 and a fusion peptide between a Tat-cell penetrating peptide and the autophagic protein beclin 1 have been shown to drastically inhibit HIV-1 replication in primary human macrophages through autophagy induction [6]. Finally, autophagy modulation has already been proposed to be a potential therapeutic target for diverse diseases, including neurodegenerative disorders and cancers. It should be noted that no deleterious effect of a chemically upregulation of autophagy *in vivo* has been reported to date [7]. In conclusion, although a lot of work is needed to better understand the relationship between autophagy and pathogens, it becomes clear that the manipulation of this process is a promising route for the elaboration of new effective treatments.
